# The Importance of Time to Prostate-Specific Antigen (PSA) Nadir after Primary Androgen Deprivation Therapy in Hormone-Naïve Prostate Cancer Patients

**DOI:** 10.3390/jcm7120565

**Published:** 2018-12-18

**Authors:** Takeshi Sasaki, Yoshiki Sugimura

**Affiliations:** Department of Nephro-Urologic Surgery and Andrology, Mie University Graduate School of Medicine, Tsu, Mie 514-8507, Japan; t-sasaki@clin.medic.mie-u.ac.jp

**Keywords:** prostate-specific antigen, androgen deprivation therapy, time to PSA nadir, fibroblasts

## Abstract

Prostate-specific antigen (PSA) is currently the most useful biomarker for detection of prostate cancer (PCa). The ability to measure serum PSA levels has affected all aspects of PCa management over the past two decades. The standard initial systemic therapy for advanced PCa is androgen-deprivation therapy (ADT). Although PCa patients with metastatic disease initially respond well to ADT, they often progress to castration-resistant prostate cancer (CRPC), which has a high mortality rate. We have demonstrated that time to PSA nadir (TTN) after primary ADT is an important early predictor of overall survival and progression-free survival for advanced PCa patients. In in vivo experiments, we demonstrated that the presence of fibroblasts in the PCa tumor microenvironment can prolong the period for serum PSA decline after ADT, and enhance the efficacy of ADT. Clarification of the mechanisms that affect TTN after ADT could be useful to guide selection of optimal PCa treatment strategies. In this review, we discuss recent in vitro and in vivo findings concerning the involvement of stromal–epithelial interactions in the biological mechanism of TTN after ADT to support the novel concept of “tumor regulating fibroblasts”.

## 1. Introduction

Androgen-deprivation therapy (ADT) is the standard initial systemic therapy for advanced prostate cancer (PCa). Generally, ADT is performed with surgical castration or pharmacologic castration (luteinizing hormone-releasing hormone agonist or antagonist) accompanied with/without an antiandrogen (combined androgen blockade: CAB). Even PCa patients with metastatic disease initially respond well to ADT, but most eventually progress to castration-resistant prostate cancer (CRPC), which has a high mortality rate. Thus, there is an urgent need for predictors of which patients are more likely to develop CRPC. Several prognostic factors identified in clinical and laboratory studies could be used to predict survival including performance status, T stage, and extent of bone metastases, as well as serum alkaline phosphatase, hemoglobin, and testosterone levels [1,2]. The J-CAPRA score is a novel, validated score encompassing initial prostate-specific antigen (PSA) levels, Gleason score, and clinical stage that can be used to predict outcomes among patients undergoing primary ADT who represent the full spectrum of risk and stage, including advanced disease [3].

PSA is currently the most useful biomarker for detection of PCa. The ability to measure serum PSA levels affected all aspects of PCa management over the past two decades. Serum PSA levels are generally proportional to tumor volume and clinical stage of the disease. Thus, despite recognized limitations, measurement of PSA is essential for screening and monitoring of treatment response, prognosis, and progression in patients with PCa [4]. We demonstrated that time to PSA nadir (TTN) after primary ADT is an important early predictor for overall survival and progression-free survival for advanced PCa patients [5,6]. Moreover, we gathered evidence from in vivo experiments to support the role of fibroblasts in the PCa tumor microenvironment in prolonging the period of serum PSA decline after ADT and enhancing the efficacy of ADT [7]. In this review, we briefly summarize the importance of TTN after primary ADT for hormone-naïve advanced PCa patients with a focus on results from both in vitro and in vivo experiments.

## 2. Prostate-Specific Antigen (PSA)

PSA is an androgen-regulated serine protease and member of the tissue kallikrein family of proteases [8]. In humans, the prostate gland consists of a single layer of secretory epithelial cells, which are surrounded by a continuous layer of basal cells and a basement membrane [9]. PSA is produced by secretory epithelial cells in the prostate gland, and is directly secreted into the prostate lumen. One characteristic of PCa is disruption of the basal cell layer and basement membrane, and this loss of normal glandular architecture results in increases in serum PSA [10]. Transcription of the *PSA* gene is normally regulated by androgens via the androgen receptor (AR) [11]. The AR is a steroid hormone receptor that binds as a homodimer to a specific DNA sequence termed the androgen-responsive element (ARE). Consensus AREs are located at −156 to −170 from the transcriptional start site of the *PSA* gene [12]. Meanwhile, the *PSA* distal enhancer is located approximately 4.2 kb upstream of the transcription start site in a region that contains a single strong consensus ARE (ARE III). The presence of multiple additional weak non-consensus AREs has also been demonstrated by binding studies showing that the cooperative binding of multiple ARs in this region likely accounts for its strong androgen-dependent activity [13,14,15,16].

## 3. PSA Expression after Androgen Deprivation Therapy (ADT)

**Figure 1:**
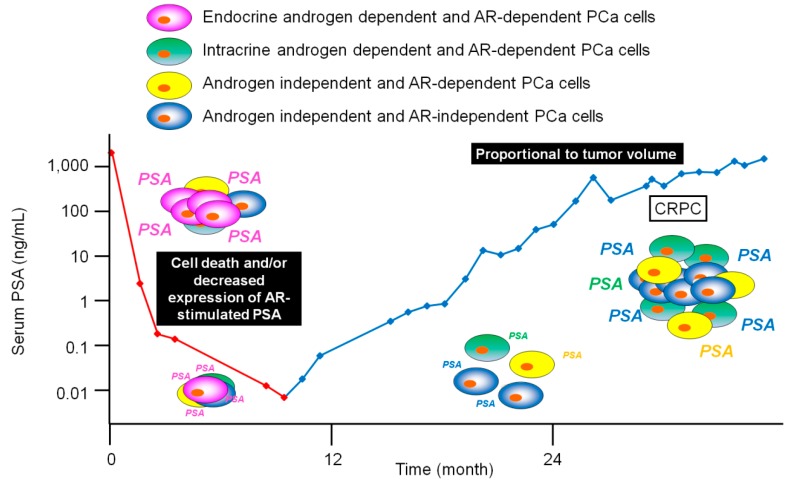
Serum PSA kinetics after primary ADT. Decreases in PSA levels following primary ADT are due to tumor cell death and/or simply decreased expression of AR-stimulated PSA in response to the absence of androgen. Figure 1 refers to [23]. AR: androgen receptor; PCa: prostate cancer; CRPC: castration-resistant prostate cancer; PSA: prostate-specific antigen; ADT: androgen-deprivation therapy.

The goal of ADT treatment for PCa patients is to downregulate concentrations of circulating androgen or block transcriptional activation of the AR [17]. The decrease in PSA levels after primary ADT most likely results from tumor cell death and/or decreased expression of AR-stimulated PSA in surviving tumor cells (Figure 1). As a result, in some cases, primary ADT may have larger effects on PSA production than on tumor survival.

In the absence of androgen, AR is activated by the protein kinase A and/or protein kinase C pathway. In LNCaP cells, androgen-independent induction of *PSA* gene expression is regulated by the AR-dependent pathway [18,19,20]. Mitogen-activated protein kinase signaling may also regulate *PSA* transcription in an androgen-independent manner [21]. A number of growth factors and cytokines, including insulin-like growth factor (IGF) 1, keratinocyte growth factor (KGF; known as fibroblast growth factor (FGF) 7), epidermal growth factor (EGF), and interleukin (IL) 6, stimulate AR signaling and PSA expression in the context of androgen deficiency [22].

## 4. Time to PSA Nadir (TTN) after Primary ADT

For PCa patients undergoing ADT, PSA kinetics are an important indicator of ADT response. However, the prognostic significance of PSA kinetics remains controversial [24]. In particular, the prognostic importance of various PSA indexes after treatment, such as PSA level at initial diagnosis, pattern of PSA decrease after hormone therapy, PSA half-life, nadir PSA level after treatment, TTN and percentage of PSA decrease, is unclear. Furthermore, few studies have examined whether these PSA indexes can accurately predict the likelihood of progression to CRPC [25]. Intuitively, most urologists expect that a more rapid PSA decline in response to primary ADT would be positively associated with extended survival. Indeed, clinical studies performed in the 1990s indicated that rapid PSA declines were associated with longer remission periods [26]. However, we have recently reported clinical evidence that a prolonged period for serum PSA decline after primary ADT was strongly indicative of disease progression in patients with advanced PCa [5,6]. Several recent studies describing results from large, multicenter investigations also demonstrated that longer TTN periods after primary ADT can predict favorable progression-free survival and overall survival in various hormone-naïve patient populations [24,27,28]. Akbay et al. evaluated PSA decline pattern after primary ADT in advanced PCa patients [29]. They showed that rapid PSA decline patients (fast decline slope) patients had higher rates of PSA progression, while prolonged PSA decline patients (slow decline slope) patients had lower rates of PSA progression. Choueiri et al. also demonstrated that higher PSA decline (≥52 ng/mL/year) was associated with shorter survival in univariate analysis [27]. These findings may seem counterintuitive in that they suggest that a more rapid response to primary ADT indicates more aggressive disease.

Here, we summarize several lines of clinical evidence showing the prognostic importance of TTN after primary ADT in hormone-naïve PCa patients (Table 1). With respect to disease progression, Morote et al. reported that a PSA nadir ≤ 0.2 ng/mL and TTN ≥ 12 months in metastatic PCa patients was associated with a low risk of PSA progression [30]. Hori et al. reported that PSA nadir < 1 ng/mL and TTN > 12 months was associated with a low risk of biochemical relapse in PCa patients with bone metastasis, whereas patients without bone metastasis with a PSA nadir < 0.1 ng/mL and TTN > 24 months had a low risk of biochemical relapse [31]. These studies could be limited by patient classification in that the study participants were stratified into two groups according to PSA nadir level or TTN. Inadequate PSA responders, with shorter TTN due to small declines in PSA from an initial high PSA level, which is obviously associated with poor progression, might have been included in the group having a shorter TTN. Interestingly, Huang et al. stratified their study participants into four groups by combining PSA nadir level and TTN. In their study, they demonstrated that patients with advanced or metastatic disease and a PSA nadir ≥ 0.2 ng/mL and TTN < 10 months had a significantly reduced disease-free progression time [32]. However, for adequate PSA responders (PSA nadir ≤ 0.2 ng/mL), they found that prolonged TTN did not correlate with longer progression-free survival due to the inclusion of patients having various clinical stages, as well as those who underwent different pretreatments, such as radical prostatectomy or radiotherapy. We propose that the optimal cut-off value for TTN be >11 months for advanced PCa patients without bone metastasis and >8 months for those with bone metastasis in both groups with ≤0.2 PSA nadir and >0.2 with and without metastasis, respectively, for progression-free survival [6]. With respect to survival, Hussain et al. reported that a PSA ≤ 4 ng/mL after 7 months of androgen deprivation is a strong and specific predictor for risk of death [33]. Choueiri et al. recently reported that a PSA nadir < 0.2 ng/mL and a TTN of > 6 months were an optimal predictor for a longer overall survival in patients with metastatic disease [27]. Our analysis showed that a PSA nadir < 0.2 ng/mL and a TTN of > 9 months were an optimal predictor of longer overall survival in patients with bone metastatic disease [5].

## 5. Biological Mechanism of TTN after ADT

The above findings indicate that a rapid decline in PSA expression after primary ADT appears to be a strong indicator of more aggressive disease. However, the mechanisms that mediate this effect remain unknown.

Generally, well-differentiated PCa cells are AR-dependent and PSA positive, whereas poorly differentiated PCa cells are AR-independent and PSA negative. Nelson et al. provided four molecular-state frameworks for AR activation in PCa after ADT—State 1: Endocrine androgen-dependent and AR-dependent; State 2: Intracrine androgen-dependent and AR-dependent; State 3: Androgen-independent and AR-dependent; State 4: Androgen-independent and AR-independent [40]. State 4 is considered to be a fatal stage in which AR signaling is abolished. The transition of PCa cells to an androgen-independent phenotype is a complex process that involves selection and outgrowth of pre-existing clones of androgen-independent cells (clonal selection) as well as adaptive upregulation of genes that promote cancer cell survival and growth after ADT (adaptation) [41]. These two mechanisms share an important prerequisite characteristic: PCa are heterogeneous tumors comprising various subpopulations of cells that respond differently to ADT [41]. Acute loss of AR function after ADT is associated not only with apoptosis and reduced PSA secretion by PCa cells, but also with triggering of AR-independent growth. Disruption of androgen signaling by ADT may inhibit cell cycle control, which could contribute to carcinogenesis [42]. Further in vitro and in vivo experiments to characterize the interaction between AR-dependent and AR-independent PCa cells will be required to confirm these clinical findings.

**Figure 2:**
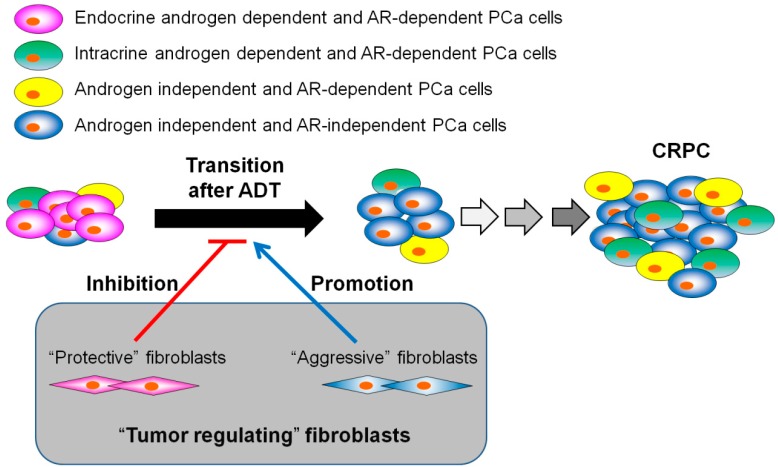
“Tumor regulating fibroblasts” and the role of fibroblasts after ADT in PCa patients. “Protective” fibroblasts inhibit the transition of PCa cells from AR-dependent to AR-independent, whereas “aggressive” fibroblasts promote the transition from AR-dependent to AR-independent [7].

We demonstrated a critical role for fibroblasts in tumor stroma in the regulation of androgen dependency of PCa cells and PSA expression after ADT [7]. The tumor stroma surrounding cancer cells is enriched in fibroblasts that secrete AR-stimulating factors, vascular endothelial growth factor (VEGF), and transforming growth factor (TGF) β [43]. Stromal–epithelial cell interactions play a crucial role in carcinogenesis and tumor progression [44]. We previously reported that stromal remodeling after castration is accompanied by changes in the expression levels of these growth factors in the prostate [45]. Importantly, most fibroblastic cells in the prostate stroma are negative for AR [46,47], and the phenotypes of human PCa fibroblastic stromal cells are broadly heterogeneous [48]. Several studies showed that that androgen-sensitive and -insensitive interactions between stromal and epithelial cells determine how prostate epithelial cells respond to androgen ablation [49,50]. In our in vitro and in vivo experiments, we found that the AR-independent and heterogeneous characteristics of fibroblasts in PCa tissue could regulate ADT efficacy as measured by tumor volume and Ki67 index, which is related to the decline in serum PSA after ADT [7]. Even though there is a limitation of animal experiments, we found that fibroblasts had two mechanisms for regulating declines in serum PSA after ADT: 1) maintenance of tumor microvessels and 2) secretion of soluble AR-stimulating factors. Fibroblasts have a diverse capacity for neovascularization and varying expression patterns of these soluble factors. In a low androgen environment, stromal–epithelial interactions may be an important mechanism to control AR activity and AR-regulated PSA expression. Thus, we advocate for the adoption of a new concept, “tumor regulating fibroblasts”, which describes the possible action of fibroblasts after ADT in PCa patients (Figure 2). Tumor-promoting “aggressive” fibroblasts, also called cancer-associated fibroblasts (CAFs), are well known [51]. CAFs surround cancer cells to support the survival and proliferation of cancer cells in a paracrine fashion. These “aggressive” fibroblasts can increase the selective pressure to promote preferential selection of more aggressive epithelial phenotypes. On the other hand, Hayashi et al. reported that rat urogenital mesenchyme, which shares similar features with CAFs, but has a physiological role that involves facilitating the development, differentiation, and, ultimately, growth quiescence of the prostate, elicited a reduction in tumorigenic potential of Dunning prostatic adenocarcinoma [52]. Recent studies also demonstrated that normal human fibroblasts can inhibit the proliferation of tumor cells [53,54]. “Protective” fibroblasts that contribute to a prolonged serum PSA decline period could enhance treatment efficacy, resulting in a more favorable prognosis.

## 6. Concluding Remarks

TTN after primary ADT for advanced PCa patients is a powerful tool for predicting disease progression and overall survival. Clarification of the mechanisms associated with TTN after primary ADT could help inform treatment decision-making to determine optimal strategies for PCa treatment. In this review, we focused on stromal–epithelial interactions to develop a clearer picture of the biological mechanism of TTN after ADT, based on findings from in vitro and in vivo experiments to provide a novel concept of “tumor regulating fibroblasts”.

## Figures and Tables

**Table 1:** Examination of TTN after primary ADT in hormone-naïve PCa patients as a prognostic marker for disease outcome.

Study	Patients (*N*)	Treatment	TTN Cutoff Threshold (Months)	Outcome
Morote et al. 2004 [30]	283 (98 locally advanced, 185 metastatic)	Orchidectomy or maximal androgen blockade	12	Progression-free survival
Morote et al. 2005 [34]	185 (metastatic)	Orchidectomy or LHRH agonist with antiandrogen	9	Progression-free survival
Choueiri et al. 2009 [27]	179 (metastatic, 47.5% had prior RP or RT)	LHRH agonist or orchidectomy with or without antiandrogen	6	Overall survival
Hori et al. 2011 [31]	155 (46 with bone metastasis, 109 without bone metastasis)	LHRH agonist or orchidectomy with or without antiandrogen	24 (without bone metastasis)	Progression-free survival
Huang et al. 2011 [32]	650 (advanced or metastatic, 35% had RP or RT)	LHRH agonist or orchidectomy with or without antiandrogen	10	Progression-free survival
Huang et al. 2011 [35]	650 (advanced or metastatic, 35% had RP or RT)	LHRH agonist or orchidectomy with or without antiandrogen	10	Overall survival
Sasaki et al. 2011 [5]	87 (with bone metastasis)	LHRH agonist or orchidectomy with antiandrogen	9	Overall survival
Sasaki et al. 2012 [6]	184 (advanced, 101 with bone metastasis, 83 without bone metastasis)	LHRH agonist or orchidectomy with antiandrogen	8 with bone metastasis, 11 without bone metastasis	Progression-free survival
Hong et al. 2012 [36]	131 (metastatic)	LHRH agonist or orchidectomy with antiandrogen	8	Progression-free survival
Zhang et al. 2013 [37]	332 (advanced or metastatic)	LHRH antagonist or orchidectomy with flutamide	10	Overall survival Progression-free survival
Kitagawa et al. 2014 [28]	10,958 (all stage)	LHRH agonist or orchidectomy with or without antiandrogen	9	Overall survival Progression-free survival
Tomioka et al. 2014 [38]	286 (metastatic)	LHRH agonist or orchidectomy with or without antiandrogen	<6, 6–12, ≥12	Overall survival Progression-free survival
Teoh et al. 2017 [39]	419 (metastatic)	LHRH agonist or orchidectomy	<3, 3–17, >17	Overall survival Progression-free survival
Akbay et al. 2017 [29]	97 (advanced)	LHRH agonist or orchidectomy with or without antiandrogen	12	Progression-free survival

LHRH: luteinizing hormone releasing hormone; RP: radical prostatectomy; RT: radiation therapy; TTN: PSA nadir; ADT: androgen-deprivation therapy; PCa: prostate cancer.
